# The unpredictable clinical course of an abdominal cyst diagnosed in the prenatal period: A case report

**DOI:** 10.1016/j.crwh.2023.e00568

**Published:** 2023-11-28

**Authors:** Carmine Sica, Giuliana Orlandi, Antonio Schiattarella, Giordana Sica, Paolo Toscano, Antonia Lettieri, Olimpia Gabrielli, Luigi Manzo, Laura Letizia Mazzarelli, Letizia Di Meglio, Lavinia Di Meglio, Ferdinando Antonio Gulino, Giosuè Giordano Incognito, Attilio Tuscano, Laura Ieno, Marco Palumbo, Maurizio Guida, Aniello Di Meglio

**Affiliations:** a Diagnostica Ecografica e Prenatale di A. Di Meglio, Naples, Italy; b Department of Neuroscience, Reproductive Sciences and Dentistry, School of Medicine, University of Naples Federico II, Naples, Italy; c Department of Woman, Child and General and Specialized Surgery, University of Campania “L. Vanvitelli”, Naples, Italy; d Radiology Department, School of Medicine, University of Milan, Milan, Italy; e Pediatric Department, Bambino Gesù Children's Research Hospital IRCCS, Rome, Italy; f Unit of Gynecology and Obstetrics, Department of Human Pathology of Adults and Developmental Age, “G. Martino” University Hospital, Messina, Italy; g Department of General Surgery and Medical Surgical Specialties, University of Catania, Catania, Italy

**Keywords:** Enteric duplication cyst, Prenatal diagnosis, Ultrasound, Magnetic resonance imaging, Congenital anomalies, Case report

## Abstract

Enteric duplication cysts are rare congenital malformations of the gastrointestinal tract. Prenatal diagnosis can be achieved through ultrasound, which may reveal a cystic mass, though the differential diagnosis is broad. We report a case in which the prenatal ultrasound detection of an abdominal cystic mass prompted postnatal magnetic resonance imaging, leading to the diagnosis of an enteric duplication cyst. At 6 weeks of age, the infant developed an obstruction of the small bowel, requiring urgent surgical intervention. This case underscores the difficulties in differentiating abdominal cysts prenatally. Thorough prenatal and neonatal follow-up is crucial, and postnatal magnetic resonance imaging is sometimes essential for accurate diagnosis. The clinical course can be unpredictable, and complications that may arise could necessitate urgent surgical treatment.

## Highlights

• The differential diagnosis for cystic abdominal masses is broad.
• A postnatal MRI may be essential for an accurate diagnosis.
• The clinical course is unpredictable, and urgent surgical treatment may be needed.

## 1 Introduction

Enteric duplication cysts are an intriguing subset of congenital anomalies of the gastrointestinal tract. They are rare, with a prevalence of approximately 1 in 4500 live births, which places them among the less commonly encountered congenital conditions within pediatric and neonatal medicine [1,2]. The cysts are typically located in the small intestine, the segment of the gastrointestinal tract that is most active in nutrient absorption and digestion. They often present a clinical enigma due to their variable size, location, and potential to communicate with the normal bowel lumen.

Notably, approximately 50% of enteric duplication cysts, are found in the mesenteric border of the ileum. This anatomical site is characterized by its rich vascular supply and complex arrangement of the bowel and mesentery, which is essential for the intricate process of nutrient absorption and intestinal mobility [3]. These cysts share a muscular wall with the ileum and are typically supplied by the same arterial blood supply that nourishes the normal bowel, suggesting a shared embryological origin that points to an error in the development of the primitive gut during the early gestational period [3].

In prenatal settings, the diagnosis is often initiated by ultrasonography (US), revealing a cystic mass, which may be characterized by its unique “double-wall” sign—a feature indicative of the cyst's layered structure [3].

However, the differential diagnosis for cystic abdominal masses detected via US i includes a wide variety of conditions, each with distinct implications for the fetus and the course of the pregnancy. Renal cysts, for example, may indicate underlying polycystic kidney disease or other nephrological disorders; choledochal cysts might necessitate postnatal surgical intervention to prevent complications like cholangitis; hepatic cysts could be a marker for a range of liver pathologies; mesenteric or omental cysts often require surgical planning for postnatal management; cystic lymphangioma might necessitate further investigation into potential systemic involvement of the lymphatic system; dilated bowel loops are a hallmark of intestinal atresia, which can be a surgical emergency after birth; and ovarian cysts in female fetuses may complicate the delivery or postnatal period [1,4].

The detection rates of enteric duplication cysts through prenatal US are relatively modest, at only 20–30%. This limitation is a concern for obstetricians and pediatric surgeons alike, as early detection can significantly influence the management plan for the infant [3,5]. The low detection rate can be attributed to factors such as the position of the fetus, maternal obesity, or the cyst's characteristics, like size and location. Moreover, the resolution of the US scan may not be sufficient to detect smaller or non-communicating cysts.

To surmount these challenges, additional imaging modalities and refined diagnostic criteria are sometimes employed. Magnetic resonance imaging (MRI) can provide high-resolution images and superior tissue characterization, potentially identifying lesions missed by US. Advanced ultrasonographic techniques, such as 3D-ultrasonography and Doppler flow studies, offer more detailed information on the cyst's structure and its vascular supply, which are invaluable in differentiating enteric duplication cysts from other cystic masses.

Despite these advanced diagnostic tools, the prognosis can be unpredictable. Some may remain asymptomatic and may even resolve spontaneously, while others can lead to complications like volvulus, intussusception, or obstruction, which require immediate medical intervention. Furthermore, the possibility of malignant transformation, although rare, adds a layer of complexity to the long-term management of these cysts.

We present a case where the prenatal detection of an abdominal cystic mass led to the performance of postnatal MRI, which enabled the diagnosis of an enteric duplication cyst. After 6 weeks, the baby developed an obstruction of the small bowel, necessitating an urgent surgical intervention.

## 2 Case Presentation

In the second trimester of a 35-year-old woman's second pregnancy, routine prenatal US screening revealed an intra-abdominal cystic mass. Her previous pregnancy had culminated in the birth of a healthy neonate via cesarean section (CS) due to the baby's breech position. Her previous medical and family histories were unremarkable. The first-trimester screening was normal, and toxoplasmosis, other agents, rubella, cytomegalovirus, and herpes simplex (TORCH) screening was negative.

The abdominal anomaly prompted a referral to a specialized center for a detailed assessment of the fetal condition.

**Fig. 1:**
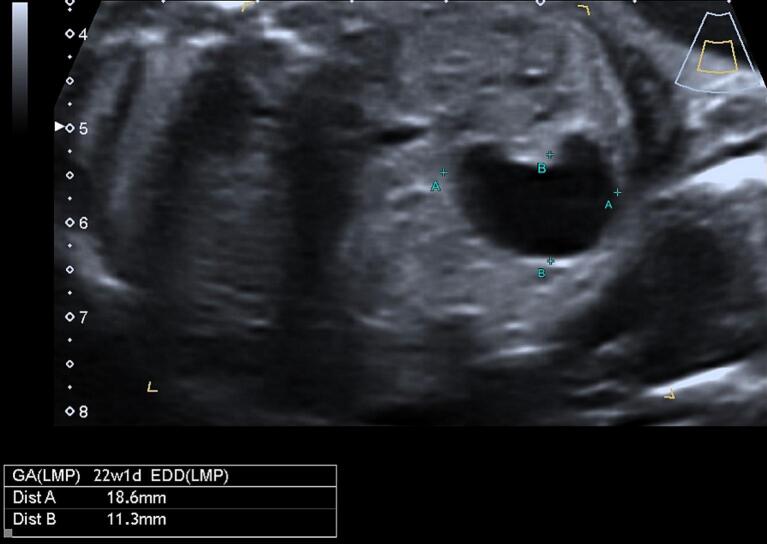
Ultrasound unilocular abdominal cystic mass image at 22 weeks of gestation.

**Fig. 2:**
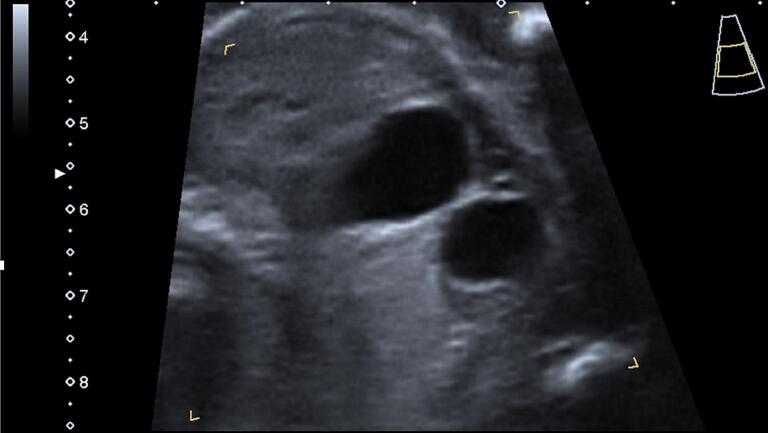
Ultrasound unilocular abdominal cystic mass image at 22 weeks of gestation separated from the stomach.

The US examination was performed with a Voluson E10 scanner (GE Healthcare Ultrasound, Milwaukee, WI, USA) equipped with a curved linear array transabdominal transducer (2–5 MHz). The fetus, at 22 weeks of gestation, exhibited normal growth parameters, with an appropriate volume of amniotic fluid and the absence of any discernible gross abnormalities. A unilocular cystic structure was identified, measuring 18 by 11 mm in the axial plane and with a third dimension of 8 mm, located in the right quadrant of the fetal abdomen. Notably, the cyst wall did not demonstrate any significant thickening or hyperechogenicity, traits often associated with certain types of abdominal cysts (Fig. 1, Fig. 2).

As the pregnancy progressed, the cyst's dimensions modestly increased—to 20 by 12 mm at 30 weeks and to 27 by 15 mm at 35 weeks. Throughout this period, vigilant monitoring was maintained to ensure the cyst did not exert undue pressure on adjacent organs.

Apart from the presence of the cyst, the pregnancy proceeded without further complications. At 39 weeks of gestation, a healthy male infant was delivered via a planned cesarean section due to the mother's previous surgical delivery and her decision against trial of labor after cesarean (TOLAC).

Initial neonatal evaluations were reassuring, and the infant had a soft abdomen with no externally palpable masses. However, a subsequent US scan detected a slightly larger cyst, measuring 20 by 25 mm, with internal echoes suggesting floating debris, situated anterior to the right kidney. Despite this finding, power Doppler sonography did not reveal any vascular signals within the mass, and there was no apparent communication with the intestinal tract.

A differential diagnosis was proposed, considering the possibilities of bowel duplication, a mesenteric cyst, or an omental cyst. To refine this diagnosis, abdominal MRI was performed on the 24th day after birth. The imaging presented a well-demarcated cystic mass adjacent to the terminal part of the small intestine, specifically near the ileocecal valve. The mass's wall was homogeneously structured, with a thickness of 2 mm, and exhibited the characteristic stratification seen in bowel tissue. The content of the cyst appeared corpuscular, yet without any signs of hemorrhage, obstruction, or continuity with the bowel wall; these findings were highly suggestive of an enteric duplication cyst.

Consultation with a pediatric surgeon was sought, but a decision was made to defer surgical intervention until the neonate attained a suitable weight for the procedure. However, on the 43rd day, the infant exhibited signs of distress, including irritability, abdominal distension, and vomiting. X-rays revealed dilated loops of the intestine, indicative of an obstruction of the small bowel.

**Fig. 3:**
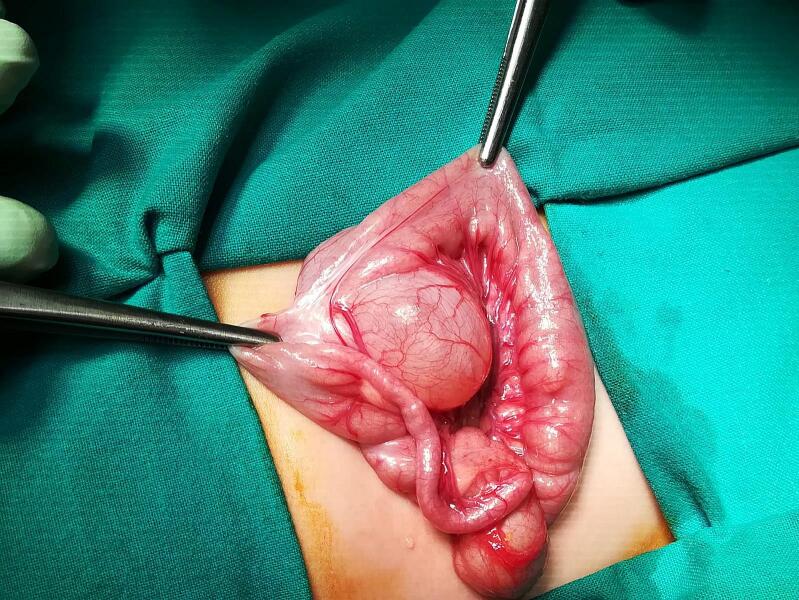
Enteric cyst before the surgical removal.

Urgent surgical exploration revealed a cystic structure, now 30 by 20 by 35 mm, attached to the mesentery of the ileum, located some 80 cm from the ileocecal valve (Fig. 3).

This cyst presented with a smooth surface of a pinkish-tan hue, and a glistening appearance. The blood vessels of the mesentery draped over the cyst, supplying both the cyst and the adjacent ileum. A significant volvulus of the ileum was discovered, characterized by a 720° counterclockwise twist. The surgical team proceeded to excise the cyst along with the involved segment of the ileum, correct the volvulus, and restore intestinal continuity via end-to-end anastomosis.

**Fig. 4:**
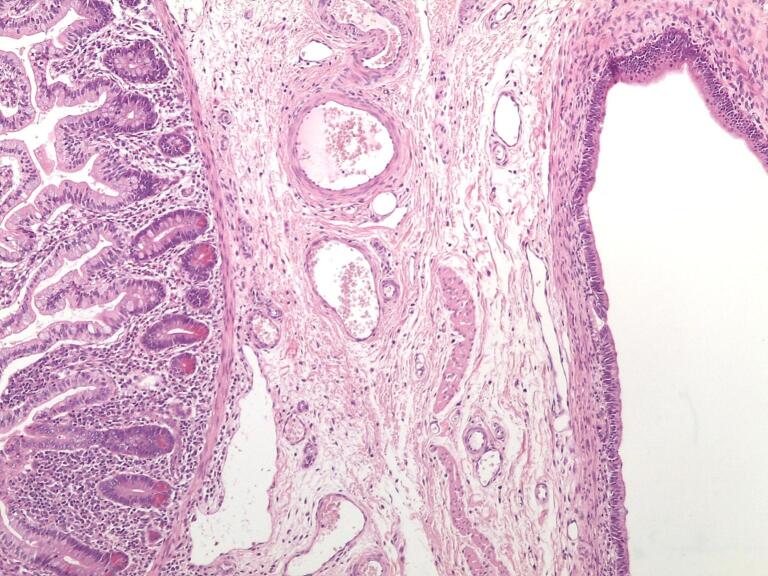
Histopathological features of the duplication; the cyst (on the right) and contiguous portion of the ileum shared a common muscular wall. Hematoxylin & eosin 200×.

Post-operative analysis confirmed the initial suspicions. The cyst was indeed unilocular, filled with a clear, mucoid fluid of a light yellowish tint. Histologically, the cyst's wall was lined with a primitive bowel mucosa and a muscular layer, partially fused with the muscular wall of the ileum (Fig. 4). These findings corroborated the diagnosis of an enteric duplication cyst.

The infant's recovery was smooth and uneventful. He began oral feeding on the fourth day after the surgery and was discharged in a healthy condition 20 days post-operatively, marking a successful resolution to a potentially complicated clinical scenario.

## 3 Discussion

Enteric duplications are a rare congenital anomaly, occurring with an estimated incidence of approximately 1 in 10,000 live births. Van Dam et al. pioneered the realm of prenatal diagnostics when they reported the first prenatal diagnosis of an enteric duplication cyst [5].

These duplications are typified by their potential to precipitate severe complications, underlining the profound importance of prenatal diagnosis. Despite its crucial role, prenatal screening presently identifies only 20–30% of lesions [3,5], marking a considerable gap in early detection.

The anatomical presence of intra-abdominal duplications is not restricted to one location; they have the potential to manifest at any point along the gastrointestinal tract. However, there is a marked predisposition for these duplications to occur within the small intestine, more specifically the terminal ileum, which accounts for over 70% of minor gastrointestinal duplications [2].

The development of enteric duplications is believed to occur during the embryonic stage of life. The precise moment of their formation is theorized to coincide with the embryonic phase of development of the alimentary tract. During this intricate process, errors such as an abnormal recanalization or a failure in the separation of the notochord and endoderm can lead to the creation of these duplicative structures. These duplications can contain a layer of smooth muscle and are lined by gastrointestinal epithelium, which is why they are referred to as “true duplications”. The complexity of this developmental mishap is compounded by the fact that these cysts can appear anywhere along the gastrointestinal tract, from the mouth to the anus, although they exhibit a predilection for the ileum [1,2].

In terms of morphology, these anomalies may present as either spherical or tubular structures. They often share a common wall with the normal gastrointestinal tract, which can be visualized using imaging techniques, where they may appear anechoic or hyperechoic. Distinguishing these duplications from other abdominal cysts such as choledochal cysts, urachal cysts, ovarian cysts, splenic cysts, renal cysts, and mesenteric cysts, or even from conditions that cause intestinal obstructions, can be challenging. The diagnostic process is aided by observing specific features like the thickness of the muscular wall and the presence of peristalsis, sometimes visible in the cystic wall of the enteric duplication, which can be pivotal in differentiating these cysts from other abdominal lesions [6].

The prenatal identification of an enteric duplication is a critical step that allows for the strategic planning of the appropriate postnatal workup. This early detection is instrumental in establishing a definitive diagnosis and in screening for associated malformations. Prenatal US is a fundamental tool for monitoring fetal development and detecting congenital anomalies [[7], [8], [9]].

Furthermore, the utility of prenatal US extends beyond initial detection; it is essential for monitoring the growth of the cyst, measuring the cyst in the plane corresponding to its maximum diameter, understanding its potential impact on surrounding structures, and planning any necessary surgical interventions after birth. However, it is a sobering reality that enteric duplications are likely to remain undetected unless the patient presents with clinical manifestations such as vomiting, abdominal distention, a palpable abdominal mass, acute intestinal obstruction, intussusception, or volvulus [2]. There are instances where patients may be entirely asymptomatic, with the duplications being incidentally discovered during routine physical examinations or through unrelated investigations such as US [10].

The variability of the US features of gastrointestinal duplications has been documented; most lesions are discerned as cystic masses that contain internal debris, and septations, and demonstrate a peristaltic motion. The wall of these cysts typically presents a double-layered appearance with a hyperechoic inner layer and a hypoechoic outer layer, mirroring the wall structure of the gastrointestinal tract [11]. Despite the characteristic appearance, clinicians are advised to exercise caution when interpreting these signs due to the documented pitfalls associated with over-reliance on the double-layered wall in diagnosing enteric duplication cysts [11]. There have been reports of artifacts that closely mimic the double-layered wall configuration of an enteric cyst [12,13], and in some instances a similar image can be observed with ovarian cysts [14,15]. Furthermore, the positioning of fetal abdominal cysts, particularly when observed in a higher location, does not categorically exclude an ovarian origin, especially if the cyst is of considerable size and could have ascended to the upper abdomen from the pelvic area due to the laxity of the fetal broad ligament [14].

In exploring the diagnostic approaches for prenatal assessment, this study primarily focused on the US. It is important to note, however, that prenatal MRI is a valuable noninvasive technique that may offer enhanced mass characterization, identify potential complications, and can be crucial in some complex cases where the US may provide limited information [16]. It can be useful also for precise preoperative assessment of these cystic masses, offering significant advantages, particularly in the identification of complications such as hemorrhages [6,15]. The findings from MRI can corroborate those from US evaluation and often provide supplementary information that can be crucial in characterizing these cysts with greater accuracy.

The cornerstone of the present work is the comprehensive description of a single case of intestinal duplication cyst using both US and MRI. This case report is instrumental in demonstrating the practical application of these imaging modalities, which could significantly enhance the management and follow-up care of patients with this condition. The ultimate aspiration is to refine prenatal diagnostic capabilities to such an extent that the onset of symptoms can be managed promptly in the early postnatal period. Nonetheless, the limitation of this study is the difficult differential diagnosis.

## 4 Conclusion

This case highlights the challenges in the differential diagnosis of abdominal cysts during the prenatal period. Postnatal abdominal MRI is sometimes necessary and pivotal in this context. The clinical course can vary, and in some instances may lead to complications that necessitate surgical intervention.
